# RNA Sequencing Reveals Phenylpropanoid Biosynthesis Genes and Transcription Factors for *Hevea brasiliensis* Reaction Wood Formation

**DOI:** 10.3389/fgene.2021.763841

**Published:** 2021-10-29

**Authors:** Xiangxu Meng, Yue Wang, Jia Li, Nanbo Jiao, Xiujie Zhang, Yuanyuan Zhang, Jinhui Chen, Zhihua Tu

**Affiliations:** ^1^ Key Laboratory of Genetics and Germplasm Innovation of Tropical Special Forest Trees and Ornamental Plants, Ministry of Education/Engineering Research Center of Rare and Precious Tree Species in Hainan Province, School of Forestry, Hainan University, Haikou, China; ^2^ Hainan Key Laboratory for Biology of Tropical Ornamental Plant Germplasm, Institute of Tropical Agriculture and Forestry, School of Forestry, Hainan University, Haikou, China; ^3^ Rubber Research Institute, Chinese Academy of Tropical Agricultural Sciences, Haikou, China; ^4^ State Centre for Rubber Breeding, Haikou, China

**Keywords:** rubber tree, rubber wood, reaction wood, phenylpropanoid biosynthesis pathway, lignin biosynthesis

## Abstract

Given the importance of wood in many industrial applications, much research has focused on wood formation, especially lignin biosynthesis. However, the mechanisms governing the regulation of lignin biosynthesis in the rubber tree (*Hevea brasiliensis*) remain to be elucidated. Here, we gained insight into the mechanisms of rubber tree lignin biosynthesis using reaction wood (wood with abnormal tissue structure induced by gravity or artificial mechanical treatment) as an experimental model. We performed transcriptome analysis of rubber tree mature xylem from tension wood (TW), opposite wood (OW), and normal wood (NW) using RNA sequencing (RNA-seq). A total of 214, 1,280, and 32 differentially expressed genes (DEGs) were identified in TW vs. NW, OW vs. NW, and TW vs. OW, respectively. GO and KEGG enrichment analysis of DEGs from different comparison groups showed that zeatin biosynthesis, plant hormone signal transduction, phenylpropanoid biosynthesis, and plant–pathogen interaction pathways may play important roles in reaction wood formation. Sixteen transcripts involved in phenylpropanoid biosynthesis and 129 transcripts encoding transcription factors (TFs) were used to construct a TF–gene regulatory network for rubber tree lignin biosynthesis. Among them, MYB, C2H2, and NAC TFs could regulate all the DEGs involved in phenylpropanoid biosynthesis. Overall, this study identified candidate genes and TFs likely involved in phenylpropanoid biosynthesis and provides novel insights into the mechanisms regulating rubber tree lignin biosynthesis.

## Introduction

The rubber tree (*Hevea brasiliensis*) is a deciduous perennial tropical tree native to the Amazon basin that produces natural rubber as well as rubber wood. Rubber wood can be used to make a variety of products, such as pulpwood for paper production and rubber wood-based panels, furniture, and joinery products (Jahan et al., 2011; Teoh et al., 2011; Severo et al., 2016). The economic importance of the rubber tree and increasing demand have promoted its widespread domestication (Priyadarshan, 2017). Given the increasing economic and application value of rubber wood, improving wood productivity and quality has become the focus of rubber tree breeding (Priyadarshan, 2017).

Wood mainly consists of cellulose, hemicellulose, and lignin. Lignin is a macromolecular biopolymer similar to cellulose, and is widely distributed in higher plants (Wang et al., 2018). During plant growth and development, lignin content increases, causing cell wall thickening and plant tissue lignification. As a crucial secondary metabolite of the phenylpropanoid biosynthesis pathway, lignin had profound effects on plant growth and development (Liu et al., 2018; Wang et al., 2019). It is extensively involved in the material transport of vascular bundles, cell wall structural integrity, stem strength, mechanical support, response to pathogens and other environmental stresses (You et al., 2013; Wang et al., 2019). Thus, the regulation of lignin synthesis affects not only lignin accumulation, but ultimately the growth and development of the entire plant.

Lignin polymers are primarily derived from the monolignols p-hydroxyphenyl, guaiacyl, and syringyl, which are formed by dehydrogenation of the hydroxycinnamyl alcohols p-coumaryl, coniferyl, and sinapyl, respectively (Zhao and Dixon, 2011). Monolignols are produced from phenylalanine through the phenylpropanoid pathway (Whetten and Sederoff, 1995). The first step of this pathway involves phenylalanine deamination by phenylalanine ammonia-lyase (PAL) to form cinnamic acid. This deamination step is followed by a series of hydroxylation reactions (catalyzed by cinnamate 4-hydroxylase, C4H; coumarate 3-hydroxylase; ferulate 5-hydroxylase, F5H; 4-coumarate-CoA ligase, 4CL; and hydroxycinnamoyltransferase, HCT), O-methylation reactions (carried out by caffeate O-methyltransferase, COMT; and caffeoyl-CoA O-methyltransferase, CCoAOMT), and reduction reactions (catalyzed by cinnamoyl-CoA reductase, CCR; and cinnamyl alcohol dehydrogenase, CAD). Lignin is ultimately formed through polymerization of monolignols by peroxidases (PODs) or laccases. The methoxylation levels of the three monolignols determine the amount and composition of lignin (Boerjan et al., 2003).

The genes for many key enzymes in lignin biosynthesis have been identified, with some also shown to influence environmental responses (Liu et al., 2018; Wang et al., 2018; Wang et al., 2019). For instance, up-regulation of *POD*, *PAL*, *C4H*, and *4CL* promotes lignin biosynthesis and increases muskmelon resistance to black spot disease (Han et al., 2018; Yan et al., 2019). Moreover, suppression of *Os4CL* expression leads to reduced lignin content in rice (*Oryza sativa*; Gui et al., 2011). Similarly, transgenic tobacco over-expressing the peroxidase gene *SWPA4* show high POD activity and lignin accumulation (Kim et al., 2008). Several transcription factors regulate the expression of lignin biosynthetic genes (Liu et al., 2018). In different plant species, TFs from the MYB (Ohtani and Demura, 2019), NAC (Ohtani and Demura, 2019), WRKY (Gallego-Giraldo et al., 2016), MADS (Li et al., 2016) and HSF (Zeng et al., 2016) families control lignin biosynthesis by regulating genes related to cell wall synthesis. These findings provided additional insight into the mechanism of lignin biosynthesis and accumulation, that is TFs could participate in lignin biosynthesis.

In this study, transcriptome sequencing of *H. brasiliensis* reaction wood uncovered genes involved in regulating lignin synthesis was firstly reported in rubber tree. Identifying transcriptome-level changes of lignin synthesis-related genes contributes to our understanding of rubber wood formation and may help elucidate the regulatory mechanisms of rubber tree lignin biosynthesis. Overall, our findings identify the candidate genes and serve as an important theoretical basis to develop breeding strategies that improve rubber tree quality.

## Materials and Methods

### Plant Material and Sample Collection

The rubber tree clones (Reyan 7-33-97) used in this study were obtained by *in vitro* tissue culture and planted in the Hainan University experimental greenhouse (Danzhou, Hainan, China; 109°29′25″ E, 19°30′40″ N) at the end of June 2016. To explore the genes involved in reaction wood formation, rubber tree trunks of similar diameter (approximately 2 cm) were selected as experimental materials. Three three-year-old rubber trees were bent at a 30° angle for 300 days to induce reaction wood formation. The bending treatment started at 9 a.m. on August 17th, 2020, and ended at 9 a.m. on June 13th, 2021. The xylem samples were collected immediately after completion of the bending treatment. Mature xylem tissue samples from NW (normal wood), TW (tension wood), and OW (opposite wood) were isolated from the same individual to enable comparisons in the same genetic background. The bark was removed from the sampling area, after which TW (upper side of the branch) and OW (lower side of the branch) were collected from the same branch using a sharp chisel, as described in Li et al. (2013). NW, which represents the control for stem xylem tissue, was isolated from the same side of the tree at breast height, approximately 1 m above the ground. All samples were approximately 2 × 1 cm and 4–5 mm deep. Samples were collected in the morning, immediately frozen in liquid nitrogen, and stored at −80°C for RNA isolation.

### RNA Extraction and Qualification

Nine samples (NW1, NW2, NW3; TW1, TW2, TW3; OW1, OW2, OW3) from 300 days rubber tree reaction wood were used for RNA extraction. A modified cetyltrimethyl ammonium bromide (CTAB) method was used to isolate total RNA according to Chang et al. (1993). Genomic DNA was eliminated by DNase treatment. RNA degradation and contamination were assessed on 1% agarose gels. A NanoPhotometer^®^ spectrophotometer (IMPLEN, CA, United States) and the RNA Nano 6000 Assay Kit of the Bioanalyzer 2,100 system (Agilent Technologies, CA, United States) were used to evaluate RNA quality and concentration for further analysis. Only RNA samples with absorption OD_260/280_ ratios from 1.9 to 2.2, OD_260/230_ ratios ≥2.0, and RNA integrity number (RIN) values greater than 6.8 were used for subsequent experiments. Polyadenylated mRNA was enriched using oligo (dT) magnetic beads.

### Transcriptome Profiling of Tension Wood, Opposite Wood, and Normal Wood From Rubber Tree Reaction Wood

For Illumina sequencing, fragmentation buffer was added to produce shorter mRNA strands. Single-stranded cDNA was synthesized from the mRNA using random hexamer primers. Double-stranded cDNA was synthesized by adding buffer, dNTPs, and DNA polymerase I. The double-stranded cDNA was purified using AMPure XP beads and subjected to end repair, addition of the poly-A tail, ligation of the sequencing linker, and fragment size selection. Finally, the nine cDNA libraries were subjected to PCR enrichment and sequenced on the Illumina HiSeq 2,500 platform.

Clean reads were obtained by removing low-quality sequence fragments caused by instrument errors, reads with low overall quality, 3’ ends with base 10 quality score (Q) < 20 (Q = −10log^error_ratio^), reads containing N blur, any adapter sequences, and any sequences with <20 nucleotides. The clean reads were aligned to the reference (ref) genome sequence (Liu et al., 2020). The read count of each gene was obtained by mapping the clean reads to the ref genome. The read counts were converted into fragments per kilobase of exon model per million mapped reads (FPKM) values.

DEGs were selected based on the following criteria: |log_2_FC| ≥ 1 and P_adjust_ (P_adj_) < 0.05. All DEGs were mapped to individual terms in the Gene Ontology (GO) database (http://www.geneontology.org/), and the number of genes per term was calculated. GO enrichment analysis was performed using GOseq software to identify significantly enriched terms. Analysis of gene regulatory pathways was conducted using the Kyoto Encyclopedia of Genes and Genomes (KEGG) Pathway database (http://www.genome.jp/kegg/pathway.html), MapMan software (v 3.6.0RC1; http://mapman.gabipd.org) was used for the functional pathway analysis (Thimm et al., 2004).

### Correlation Networks and Promoter Analysis

Co-expression among genes and TFs was assessed based on the Pearson correlation coefficient calculated in R (version 4.0.1) (Supplementary Table S1). The TF–gene interaction networks were visualized using Cytoscape (version 3.7.2; Shannon et al., 2003). Rubber tree gene promoter sequences (2000 bp upstream of the transcription start site in most cases) were retrieved and analyzed. Promoter analysis and prediction of TF binding sites was performed using PlantPAN 3.0 (http://plantpan.itps.ncku.edu.tw/; Chow et al., 2019).

### Lignin Quantification

Quantification of lignin content was performed as in Xiong et al. (2005). The sample (1 g) was crushed and then 0.1 g was added into a centrifuge tube. Subsequently, 10 ml of 1% acetic acid solution was added; the sample was mixed and centrifuged at 4,500 rpm for 5 min. The resulting precipitate was washed with 5 ml of 1% acetic acid, followed by addition of 3–4 ml of ethanol and ether mixture (1:1). The mixture was soaked at room temperature for 3 min, and the supernatant was discarded for 3 times. The precipitate in the centrifuge tube was evaporated in a boiling water bath, and 3 ml of 72% sulfuric acid was added to the precipitate. The precipitate was stirred well with a glass rod, and cellulose was dissolved by incubating the sample at room temperature for 16 h. Then, 10 ml distilled water was added to the test tube, stirred well with a glass rod, and placed in a boiling water bath for 5 min. After cooling, 5 ml of distilled water and 0.5 ml of 10% barium chloride solution were added, mixed well, and centrifuged. The precipitate was washed twice with distilled water, and 10 ml of 10% sulfuric acid and 10 ml of 0.1 mol/L potassium dichromate solution were added to the washed lignin precipitate. The test tube was placed in a boiling water bath for 15 min, stirring constantly during the process, and allowed to cool for later use.

All materials from the cooled test tube were transferred to a beaker for titration and the remaining material was washed with 15–20 ml distilled water. Then, 5 ml of 20% KI solution and 1 ml of 0.5% starch solution were added to the beaker and titrated with 0.2 mol/L sodium thiosulfate. Note: Separate titration and add 10 ml of 10% sulfuric acid and 10 ml of 0.1 mol/L potassium dichromate solution as a blank sample.

Lignin content was calculated using the following equation: % lignin $=\frac{\text{k}\left(\text{a}-\text{b}\right)}{\text{n}\times 48}$ , where 48 is the 1 mol of C_11_H_12_O_4_ equivalent to sodium thiosulfate, k is the concentration of sodium thiosulfate (mol/L), a is the volume of sodium thiosulfate consumed in blank titration (ml); b is the volume of sodium thiosulfate consumed by the titration solution (ml); n is the mass of the sample (g).

### Validation of Gene Expression by Reverse-Transcription Quantitative PCR (RT-qPCR)

cDNA was synthesized by reverse transcription using 1 μg total RNA from the nine samples (NW1, NW2, NW3; TW1, TW2, TW3; OW1, OW2 and OW3), respectively. Primer Premier v5 software was used to design primers specific to the selected genes (Table 1). Five genes were randomly selected from the NW, TW, and OW of rubber tree reaction wood. For RT-qPCR analysis, TB Green Premix Ex Taq II (Tli RNaseH Plus; Takara, Beijing, China) was used following the manufacturer’s recommendations. PCR amplification was performed in a preheated (94°C) thermal cycler and incubated at 94°C for 2 min, followed by 40 cycles of 95°C for 5 s and 60°C for 30 s. For melting curve analysis, samples were denatured at 95°C for 15 s, then cooled to 60°C (4°C per second). Fluorescence signals were collected at 520 nm continuously from 60°C to 95°C (0.2°C per second). Expression stability of the *40S* (Lertpanyasampatha et al., 2014; Pramoolkit et al., 2014) and *Ubiquitin* (Li et al., 2011; Chao et al., 2016) rubber tree genes has been previously evaluated and both were confirmed as suitable internal control genes. Thus, these two genes served as internal controls for normalization. Expression levels of the DEGs were calculated using the 2^−△△Ct^ method against the internal control genes (Schmittgen and Livak, 2008). Three technical replicates per sample were analyzed to ensure reproducibility and reliability.

**TABLE 1:** Oligonucleotide primers used for RT-qPCR in this study.

Gene id	Forward primer	Reverse primer
*Ubiquitin*	ATG​CAG​CAC​CGG​GAA​TAA​GT	GGT​CAT​CAG​GGT​TTG​GAG​CA
*40S*	TGC​AGA​ACG​AAG​AGG​GTC​AG	TTG​TTG​GCG​GAG​TTC​AAC​CT
gene-GH714_003817	GCT​AAT​GTC​ACG​GGC​TCC​AT	TGG​GAT​CGA​CCC​AGA​TAA​CG
gene-GH714_035841	ATT​CTG​GGT​CTG​GTC​TCC​CA	CCC​CTC​CCC​AAT​TCG​GTT​TT
gene-GH714_043603	GCG​AAA​TCG​CTC​CGA​TCA​AC	CGA​GTC​ACT​CTG​GGT​TCC​AC
gene-GH714_019256	GCT​CAT​GTG​GCT​TTC​ATG​GC	GAA​CCC​TTC​CCC​TTG​CCT​AC
gene-GH714_027628	GGT​GAG​CAC​ATG​CCT​TGT​TG	GCC​CTG​AGT​GAA​CGA​AGT​GA

## Results

### Global Transcriptome Analysis of RNA-Seq Data

**FIGURE 1:**
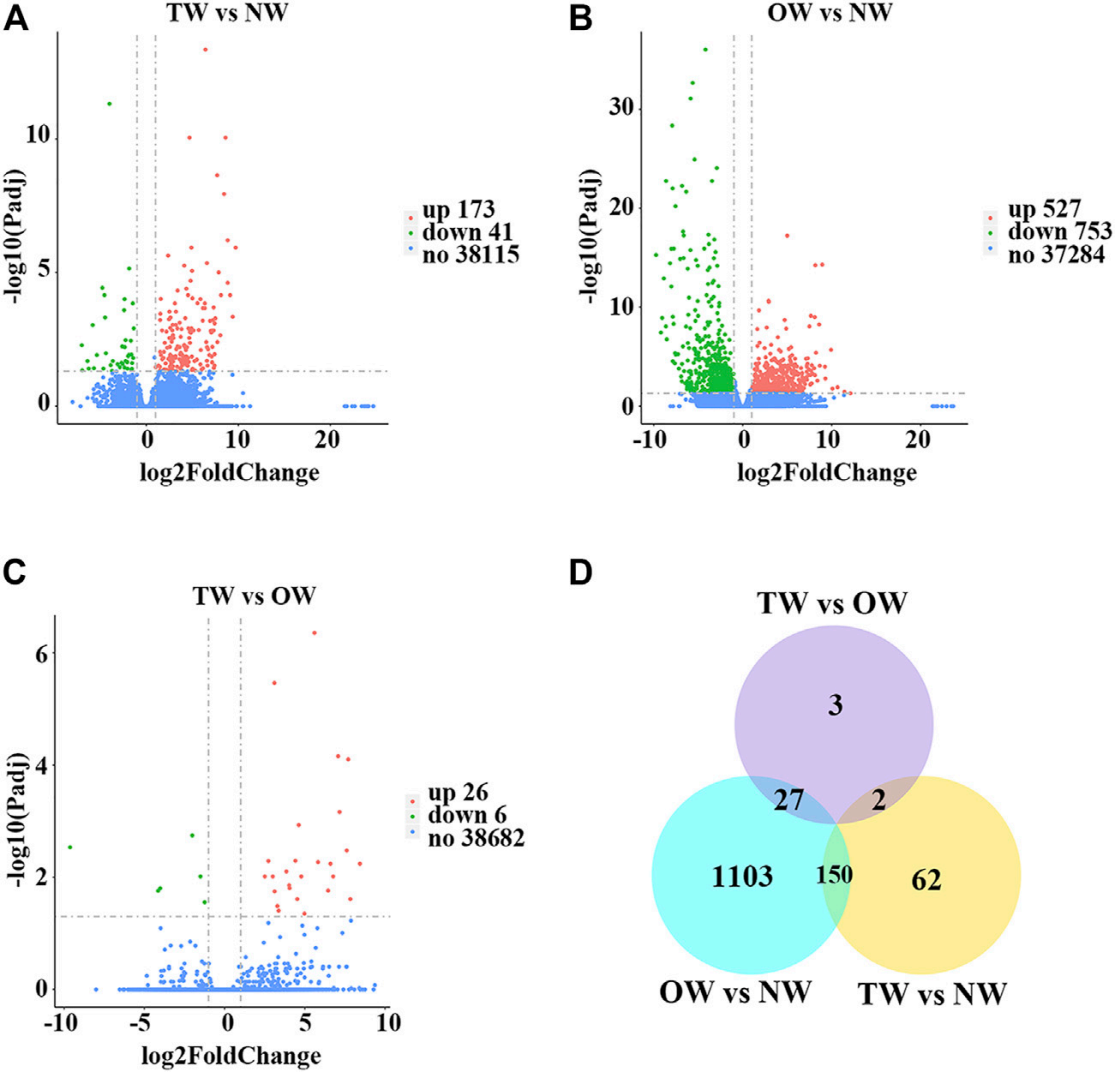
Global analysis of gene expression in different xylem tissues of rubber tree reaction wood. Volcano plots illustrate differentially expressed genes identified in TW vs. NW **(A)**, OW vs. NW **(B)**, and TW vs. OW **(C)** as individual dots. Red represents up-regulation and green represents down-regulation. **(D)** Venn diagram showing the total number of differentially expressed genes identified in TW vs. NW, OW vs. NW, and TW vs. OW, and overlap among these comparison groups.

To evaluate whether the RNA-seq data were sufficient for further analysis, we first assessed their global quality. The RNA-seq experiment generated a total of 125,970,348 (TW), 127,449,704 (OW), and 126,820,416 (NW) raw reads. After trimming, 123,519,666 (TW), 124,605,772 (OW), and 124,379,132 (NW) clean reads remained (Table 2). Among the total clean reads, 115,335,701 (TW), 116,188,884 (OW), and 115,545,396 (NW) were mapped to the rubber tree genome with mapping ratios of 93.37% (TW), 93.26% (OW), and 92.90% (NW) (Table 2). Based on previous studies (Geraldes et al., 2011; Chen et al., 2015a), these results indicated that our RNA-seq results detected most expressed genes were sufficient for subsequent quantitative analysis. To measure changes in gene expression and find key genes involved in reaction wood formation, we further selected DEGs that met the following statistical significance criteria: |log_2_FC| ≥ 1 and P_adj_ ≤ 0.05. In total, 214 (TW vs. NW; 173 up-regulated and 41 down-regulated), 1,280 (OW vs. NW; 527 up-regulated and 753 down-regulated), and 32 (TW vs. OW; 26 up-regulated and 6 down-regulated) DEGs were identified in rubber tree reaction wood (Figures 1A–C). After removing repetitive genes, this analysis identified 1,347 genes that were significantly differentially expressed in the TW, OW and NW xylem tissues (Figure 1D). The DEGs obtained from three comparison groups were used for subsequent analysis.

**TABLE 2:** Summary of RNA-seq data from normal (NW), tension (TW), and opposite wood (OW).

Sample	Raw reads	Clean reads	Mapped to genome	Mapping ratio (%)
TW1	43,083,708	42,256,214	39,392,727	93.22
TW2	40,537,540	39,876,902	37,237,975	93.38
TW3	42,349,100	41,386,550	38,704,999	93.52
OW1	40,941,484	40,039,822	37,388,130	93.38
OW2	44,907,906	43,987,054	40,848,282	92.86
OW3	41,600,314	40,578,896	37,952,472	93.53
NW1	42,924,560	41,990,498	38,750,485	92.28
NW2	41,406,942	40,674,052	37,794,460	92.92
NW3	42,488,914	41,714,582	39,000,451	93.49

### GO and KEGG Enrichment Analyses of DEGs That Participate in Reaction Wood Formation

RNA-seq provided an overview of genes that are differentially expressed during reaction wood formation. To better understand the function of these DEGs and their associated biological processes, GO and KEGG enrichment analyses were carried out for the different comparison groups shown in Figure 1D.

**FIGURE 2:**
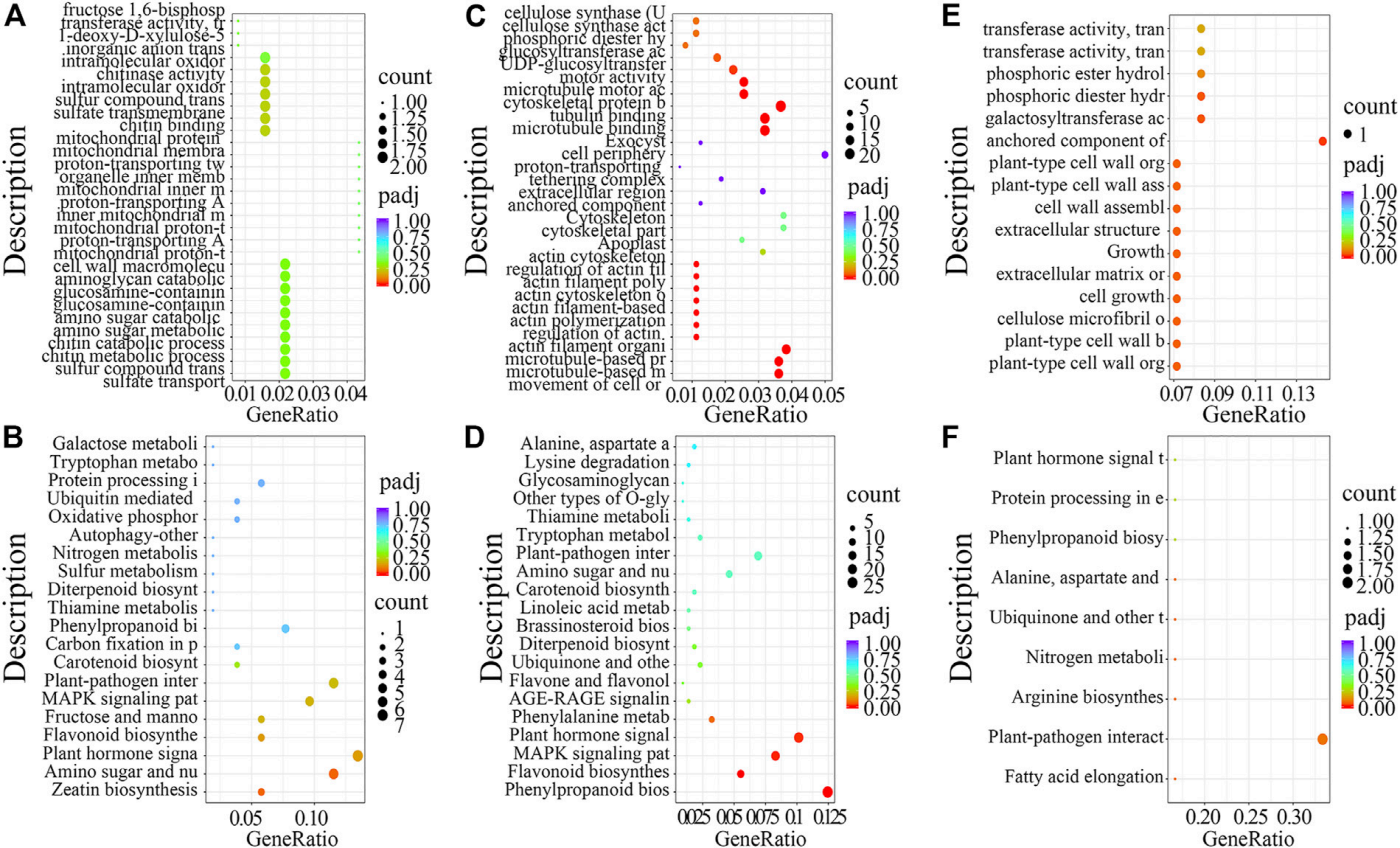
GO and KEGG enrichment analysis of differentially expressed genes (DEGs) from the RNA-seq dataset. GO analysis of DEGs identified in TW vs. NW **(A)**, OW vs. NW **(C)**, and TW vs. OW **(E)**. KEGG analysis of DEGs identified in TW vs NW **(B)**, OW vs NW **(D)**, and TW vs OW **(F)**, respectively.

First, for DEGs in TW vs. NW, GO analysis revealed enrichment for intramolecular oxidoreductase activity, cell wall macromolecule metabolic process, and sulfate transmembrane transporter activity (Figure 2A), while KEGG enrichment analysis revealed that zeatin biosynthesis and plant hormone signal transduction pathways were significantly enriched (Figure 2B). Next, GO analysis of the DEGs from OW vs. NW showed that biological processes including cellulose synthase (UDP-forming) activity, cellulose synthase activity, and UDP-glucosyltransferase activity were significantly enriched (Figure 2C), and KEGG analysis showed that phenylpropanoid biosynthesis and plant hormone signal transduction pathway were significantly enriched (Figure 2D). Last, GO analysis of the DEGs from TW vs. OW showed that cell growth, cell wall assembly, and cellulose microfibril organization were significantly enriched (Figure 2E). KEGG analysis of DEGs from TW vs. OW showed enrichment for plant–pathogen interaction pathway (Figure 2F).

Taken together, results of the GO and KEGG analyses of DEGs from different treatment groups suggest that zeatin biosynthesis, plant hormone signal transduction, phenylpropanoid biosynthesis, and plant–pathogen interaction pathway might function in the formation of reaction wood.

### Expression Analysis of DEGs Involved in Phenylpropanoid Biosynthesis and Lignin Quantification

**FIGURE 3:**
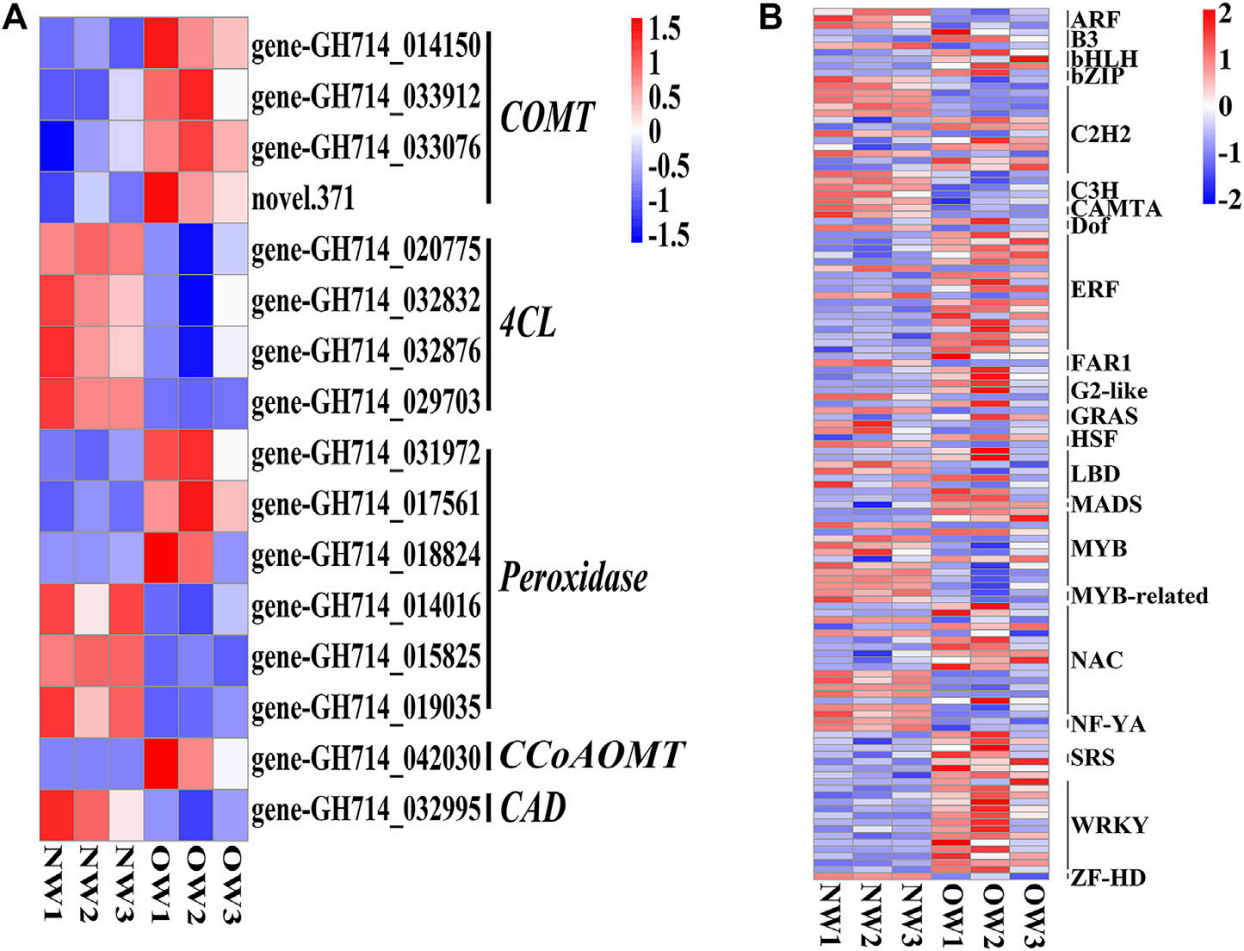
Differentially expressed genes (DEGs) and transcription factors in OW vs. NW. Heatmap showing the expression of the DEGs **(A)** and transcription factors **(B)** identified in OW vs. NW. Columns represent three different samples per tissue type and rows represent different transcripts. Each square represents a transcript and the color indicates the level of expression; red represents up-regulation and blue represents down-regulation.

**FIGURE 4:**
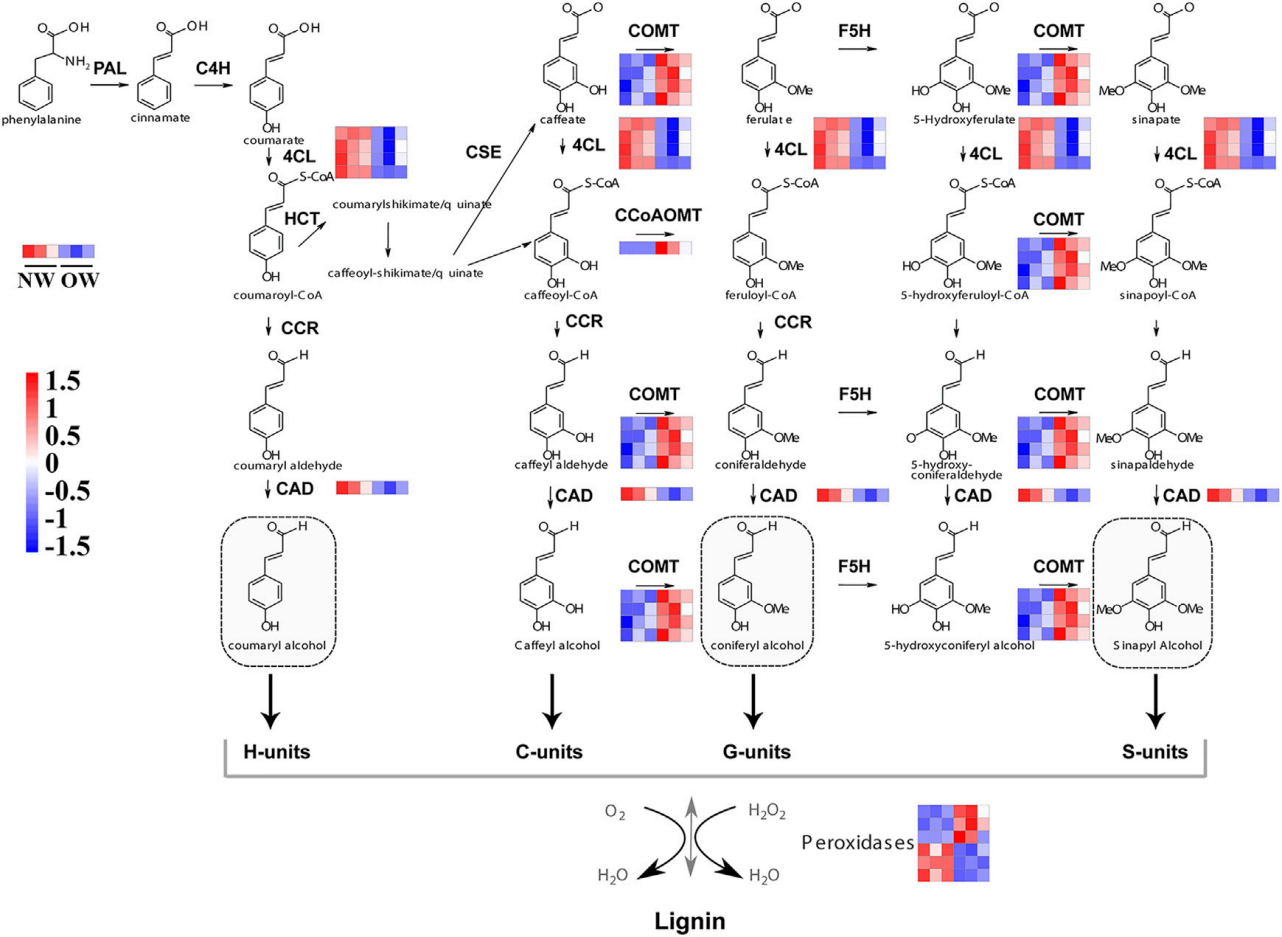
Expression of genes in phenylpropanoid biosynthesis pathway in OW vs. NW. Red represents up-regulation and blue represents down-regulation. PAL, Phenylalanine ammonia-lyase; C4H, Cinnamate 4-hydroxylase; 4CL, 4-coumarate-CoA ligase; F5H, Ferulate 5-hydroxylase; CCoAOMT, Caffeoyl-CoA O-methyltransferase; CCR, Cinnamoyl-CoA reductase; CAD, Cinnamyl alcohol dehydrogenase; COMT, Caffeate O-methyltransferase; HCT, hydroxycinnamoyltransferase; POD, peroxidase.

The phenylpropanoid biosynthesis pathway is responsible for lignin production and thus plays an important role in wood formation. KEGG enrichment analysis indicated that DEGs from OW vs. NW are involved in the phenylpropanoid biosynthesis pathway. A total of 16 transcripts of five genes from OW vs. NW are related to lignin synthesis (Figure 3A). The *4CL* gene was down-regulated in OW; 4CL converts coumarate, caffeate, ferulate, 5-hydroxyferulate, and sinapate to coumaroyl-CoA, caffeoyl-CoA, feruloyl-CoA, 5-hydroxyferuloyl-CoA, sinapoyl-CoA (Figure 4). The *CAD* gene was also down-regulated; CAD converts coumaryl aldehyde, caffeyl aldehyde, coniferaldehyde, 5-hydroxy-coniferaldehyde, and sinapaldehyde to coumaryl alcohol, caffeyl alcohol, coniferyl alcohol, 5-hydroxyconiferyl alcohol, and sinapyl alcohol (Figure 4). Down-regulation of *4CL* and *CAD* in OW vs. NW may imply that these two conversion steps are suppressed in reaction wood formation.

The *COMT* and *CCoAOMT* genes were significantly up-regulated in OW vs. NW. These two genes are involved in eight steps of lignin synthesis, including in the conversion of caffeyl alcohol to coniferyl alcohol and 5-hydroxyconiferyl alcohol to sinapyl alcohol (Figure 4). Up-regulation of *COMT* and *CCoAOMT* would promote the synthesis of G- and H-lignin (Figure 4), potentially leading to higher lignin content in OW than NW. Furthermore, three out of six transcripts encoding peroxidases were significantly up-regulated in OW, while the other three were significantly up-regulated in NW (Figure 4). These results suggest that changes in *POD* gene expression may not affect lignin content.

To link the gene expression data with effects on lignin synthesis, lignin levels were quantified in NW, TW, and OW. The average lignin content of each tissue was 263.54 mg/g in OW, 248.68 mg/g in NW, and 240.06 mg/g in TW. There were no statistically significant differences detected in the lignin content of these different tissues (Table 3). These results are consistent with the changes in expression levels of DEGs in the phenylpropanoid biosynthesis pathway.

**TABLE 3:** Lignin content of normal (NW), tension (TW), and opposite wood (OW).

Sample	Lignin (mg/g)
NW1	228.46
NW2	267.66
NW3	249.91
TW1	244.59
TW2	235.87
TW3	239.72
OW1	276.27
OW2	258.26
OW3	256.09

### Transcription Factors Mediated Transcriptional Regulatory Networks Involved in Phenylpropanoid Biosynthesis

DEGs encoding TFs in each comparison group were identified to discover potential transcriptional regulatory networks in phenylpropanoid biosynthesis (Figure 3B). In total, 129 TFs and 16 DEGs associated with phenylpropanoid biosynthesis from OW vs. NW were used to construct a co-expression network using Pearson correlation coefficients.

**FIGURE 5:**
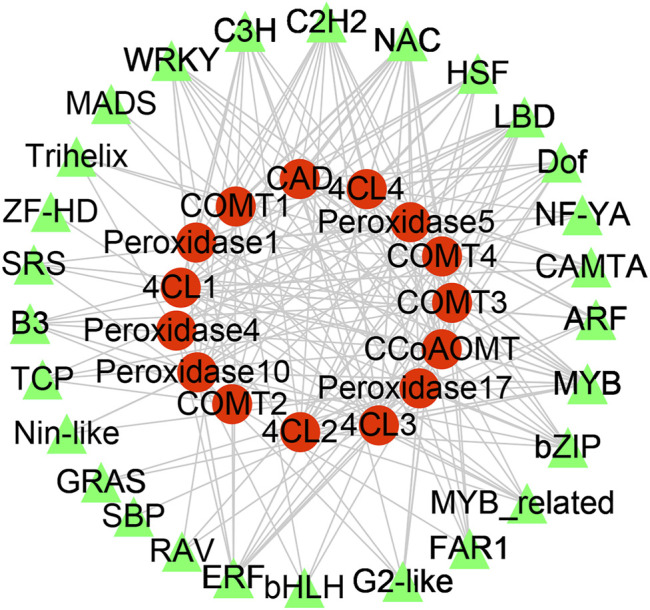
Transcription factor-gene network in rubber tree lignin biosynthesis. Red circles represent genes and green triangles represent transcription factors.

Significantly co-expressed TF–gene pairs (|cor| ≥ 0.9 and *p* < 0.05) were selected to construct the transcriptional regulatory network (Figure 5). In the network, 27 TF families regulate phenylpropanoid biosynthesis-related DEGs. *COMT*, *4CL*, *POD*, *CCoAOMT*, and *CAD* were regulated by 20, 20, 24, 10, and 12 TF families, respectively (Figure 5). Notably, the MYB, C2H2, C3H, and NAC families could regulate all the DEGs involved in phenylpropanoid biosynthesis (Figure 5). We evaluated the promoter sequence of genes co-expressed with TFs for potential TF binding sites. Promoter analysis showed that MYB, C2H2, and NAC TF families could bind to the promoter sequence of all DEGs involved in phenylpropanoid biosynthesis (Supplementary Table S2). These results suggest that these three TF families are likely key players in rubber tree reaction wood formation by regulating numerous downstream genes involved in phenylpropanoid biosynthesis.

### Validation of RNA-Seq Gene Expression Data by RT-qPCR

**FIGURE 6:**
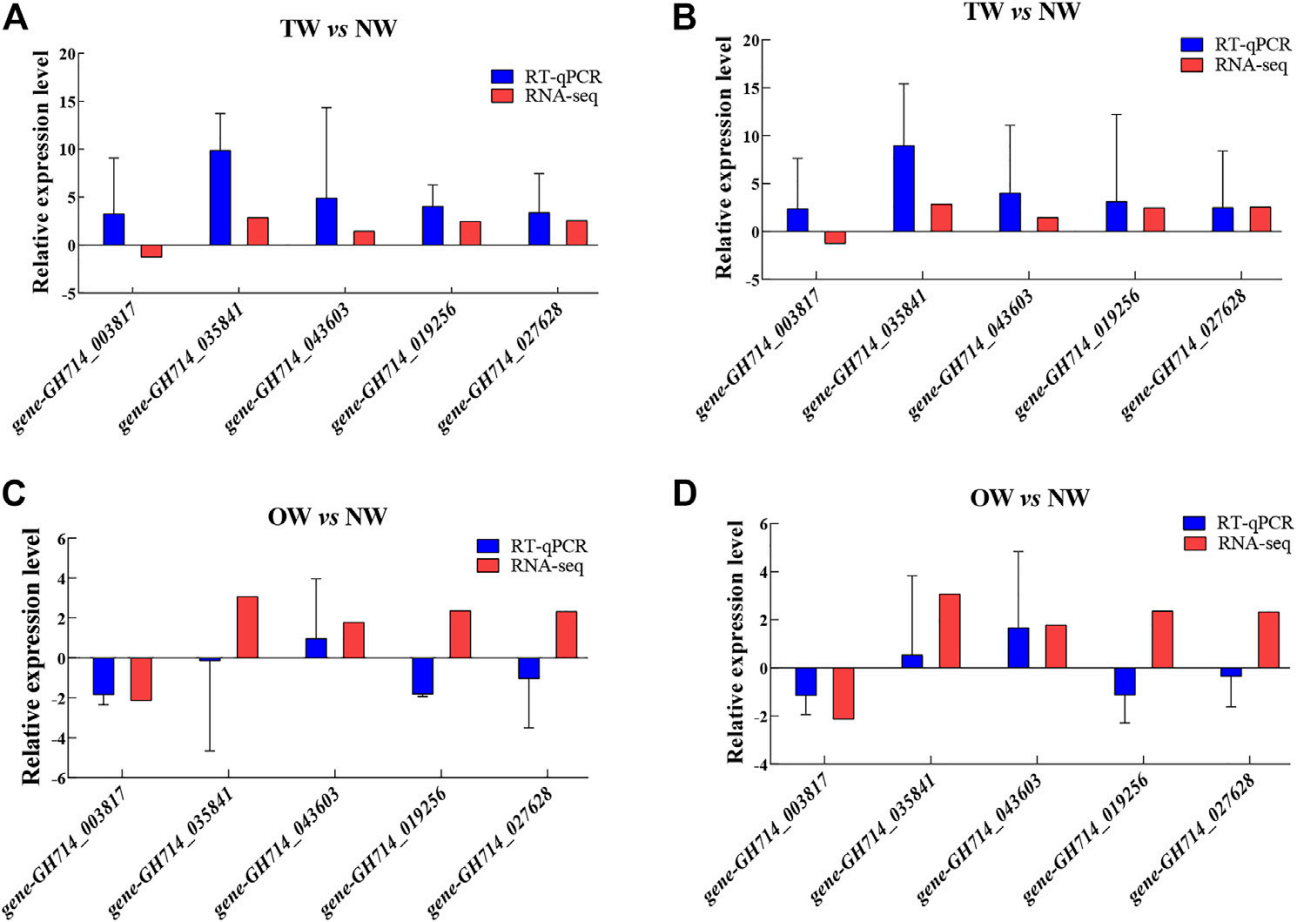
Relative expression of five genes in TW vs. NW and OW vs. NW. Expression levels were determined by RT-qPCR using the *40S* gene **(A and C)** or the *Ubiquitin* gene **(B and D)** as the internal control for normalization. Error bars indicate standard deviation of three biological replicates.

The expression patterns of most genes in the NW, TW and OW showed similar trends between the high-throughput RNA-seq data and RT-qPCR data (Figure 6). Although the relative expression level calculated by sequencing did not exactly match the expression values detected by RT-qPCR, the expression profiles were mostly consistent for the genes tested. These results confirm the reliability of the gene expression values generated from the RNA-seq data.

## Discussion

### A Model for Reaction Wood Formation Identifies Genes Involved in Wood Formation of Rubber Tree and Other Tree Species

To explore the mechanisms of rubber tree reaction wood formation, wood formation-related genes were identified and their expression patterns were investigated. In this study, we identified 214, 1,280, and 32 DEGs from three comparison groups (TW vs. NW, OW vs. NW, and TW vs. OW), respectively (Figure 1). Among them, 62, 1,103, and 3 DEGs were unique to TW vs. NW, OW vs. NW, and TW vs. OW, respectively. There were no DEGs common to the three comparison groups (Figure 1D), which might indicate that these tissue-specific DEGs function in wood formation of different xylem tissues. In addition, GO and KEGG enrichment analyses showed that the DEGs from different comparison groups have unique functions and participate in different pathways (Figure 2), further confirming that these DEGs perform distinct functions in different xylem tissues.

Based on the GO and KEGG enrichment analyses, our subsequent work focused on genes involved in phenylpropanoid biosynthesis, as well as TFs identified in the list of DEGs from OW vs. NW (Figure 2D, Figure 3). The heatmap and interaction network showed that phenylpropanoid biosynthesis-related genes and TFs had divergent expression patterns in OW vs. NW, which may reflect the diverse functions of these genes and the complexity of the regulatory network associated with wood formation (Figures 3, 5). These results further demonstrate that wood development is a complex biological process. Genes involved in wood formation may be effectively identified through our model of reaction wood formation.

Previous research has shown that many factors are involved in reaction wood formation, such as non-coding RNA, single nucleotide polymorphisms etc. Xiao et al. (2020) previously reported that long non-coding RNAs (lncRNAs) might participate in *Catalpa bungei* tension wood formation by regulating genes involved in indoleacetic acid (IAA) and abscisic acid (ABA) synthesis. Similarly, microRNA (miRNA)- and lncRNA-mediated regulatory networks widely participate in reaction wood formation of *Populus tomentosa (Pto)*, and single nucleotide polymorphisms of miRNAs and their target genes also influence this process (Chen et al., 2015b; Chen et al., 2016). Moreover, Liu et al. (2021) induced tension wood in stems of black cottonwood (*Populus trichocarpa*) and established a transcriptional regulatory network in which *PtrHSFB3-1* and *PtrMYB092* directly activate eight and 11 monolignol genes that participate in reaction wood formation, respectively. Future research should focus on characterizing the roles of non-coding RNAs and the candidate genes identified through our RNA-seq analysis in rubber tree reaction wood formation.

### Characterization of Genes Associated With Phenylpropanoid Biosynthesis in Rubber Tree by Transcriptome Analysis

Lignin synthesis is positively associated with the expression levels of *4CL*, *COMT*, *CCoAOMT*, *CAD*, and *POD* (Do et al., 2007; Vanholme et al., 2008; Wagner et al., 2011; Hu et al., 2017; Chanoca et al., 2019). In this study, *COMT* and *CCoAOMT* were up-regulated in OW vs. NW, suggesting that the lignin biosynthetic process may be enhanced in this tissue. However, *4CL* and *CAD* were down-regulated, indicating that the reaction steps involving *4CL* and *CAD* may be inhibited in OW versus NW. Moreover, three *peroxidase* gene transcripts were up-regulated in OW, while three were up-regulated in NW. This indicates that *peroxidase* genes likely generate different transcripts in different rubber tree xylem tissues to maintain lignin biosynthesis.

Previous studies showed that repressing steps of the lignin biosynthetic pathway, from PAL to CAD, may lead to reduced lignin content (Chanoca et al., 2019). In addition, studies have shown that single or double Arabidopsis knockout mutants in several *peroxidase* genes typically exhibit minor but noticeable reductions in lignin content and/or altered lignin composition in inflorescence stems, suggesting that changes in *peroxidase* expression levels may not strongly affect lignin synthesis (Herrero et al., 2013; Barros et al., 2015). Thus, changes in *peroxidase* gene levels observed in this work likely did not affect lignin content between OW and NW. Overall, the lack of a significant difference in lignin content among the different tissue types in this study may be explained by the opposite expression patterns of *4CL*, *COMT*, *CCoAOMT*, *CAD*, and by the minor contribution of the *peroxidase* genes to lignin levels.

### NAC and MYB Transcription Factor Families Participate in Rubber Tree Lignin Synthesis

Wood formation is an essential yet complex biological process arising from plant secondary growth. Important transcription factors involved in secondary growth have been identified, such as members of the MYB and NAC TF families (Demura and Fukuda, 2007; Zhong and Ye, 2009). In this study, 129 TFs from 27 TF families were responsive to reaction wood treatment (Figures 3, 5). Among them, TFs from the MYB and NAC families could regulate all DEGs involved in phenylpropanoid biosynthesis and might play pivotal roles in rubber tree reaction wood formation. Previous studies have shown that overexpression of MYB transcription factors *PtoMYB92*, *PtoMYB216*, and *PtoMYB74* could up-regulate genes involved in lignin biosynthesis and promote the formation of additional xylem layers, thicker xylem cell walls, as well as ectopic lignin deposition; these overexpression plants accumulated 13–50% more lignin (Tian et al., 2013; Li et al., 2015; Li et al., 2018). Similarly, overexpression of *NAC141* in Arabidopsis resulted in enhanced expression of lignin biosynthesis genes, stronger lignification, larger xylem, and higher lignin content compared with wild-type plants (Sun et al., 2021). These findings support the TF–gene regulatory network for rubber tree wood formation generated in this study, in which MYB and NAC TFs likely play important roles.

## Conclusion

Identification of DEGs through RNA-seq analysis on mature xylem from TW, OW, and NW of rubber tree reaction wood provided insight into the molecular basis of lignin biosynthesis. Our work demonstrated that zeatin biosynthesis, plant hormone signal transduction, phenylpropanoid biosynthesis, and plant–pathogen interaction pathways likely influence reaction wood development. A transcriptional regulatory network analysis revealed that three TF families (MYB, C2H2, and NAC) could regulate all DEGs involved in phenylpropanoid biosynthesis and play important roles in lignin biosynthesis for rubber tree reaction wood formation.

Taken together, the findings presented here advance our understanding of the regulation of phenylpropanoid biosynthesis, a critical pathway for lignin production in perennial trees. The publicly available transcriptomic dataset provides essential information for further transcriptomic, genomic, and functional genomics research in rubber tree. Overall, the results generated in this study will serve as an important resource for future studies on this economically important tropical tree crop.

## Data Availability

The transcriptome data for 300-day rubber tree reaction wood reported in this paper has been deposited at the Genome Sequence Archive (Wang et al., 2017) in BIG Data Center (BIG Data Center Members, 2019), Beijing Institute of Genomics (BIG), Chinese Academy of Sciences, under accession numbers CRA004818, and are publicly available at https://bigd.big.ac.cn/gsa.

## References

[B1] Barros J. Serk H. Granlund I. Pesquet E. (2015). The Cell Biology of Lignification in Higher Plants. Ann. Bot. 115, 1053–1074. 10.1093/aob/mcv046 25878140 PMC4648457

[B2] Boerjan W. Ralph J. Baucher M. (2003). Lignin Biosynthesis. Annu. Rev. Plant Biol. 54, 519–546. 10.1146/annurev.arplant.54.031902.134938 14503002

[B3] Chang S. Puryear J. Cairney J. (1993). A Simple and Efficient Method for Isolating RNA from Pine Trees. Plant Mol. Biol. Rep. 11, 113–116. 10.1007/bf02670468

[B4] Chanoca A. De Vries L. Boerjan W. (2019). Lignin Engineering in Forest Trees. Front. Plant Sci. 10, 912. 10.3389/fpls.2019.00912 31404271 PMC6671871

[B5] Chao J. Yang S. Chen Y. Tian W.-M. (2016). Evaluation of Reference Genes for Quantitative Real-Time PCR Analysis of the Gene Expression in Laticifers on the Basis of Latex Flow in Rubber Tree (*Hevea Brasiliensis* Muell. Arg.). Front. Plant Sci. 7, 1–9. 10.3389/fpls.2016.01149 27524995 PMC4965454

[B6] Chen J. Chen B. Zhang D. (2015a). Transcript Profiling of *Populus Tomentosa* Genes in normal, Tension, and Opposite wood by RNA-Seq. BMC Genomics 16, 1–16. 10.1186/s12864-015-1390-y 25886950 PMC4372042

[B7] Chen J. Quan M. Zhang D. (2015b). Genome-wide Identification of Novel Long Non-coding RNAs in *Populus Tomentosa* Tension wood, Opposite wood and normal wood Xylem by RNA-Seq. Planta 241, 125–143. 10.1007/s00425-014-2168-1 25230698

[B8] Chen J. Xie J. Chen B. Quan M. Li Y. Li B. (2016). Genetic Variations and miRNA-Target Interactions Contribute to Natural Phenotypic Variations in *Populus* . New Phytol. 212, 150–160. 10.1111/nph.14040 27265357

[B9] Chow C.-N. Lee T.-Y. Hung Y.-C. Li G.-Z. Tseng K.-C. Liu Y.-H. (2019). Plantpan3.0: A New and Updated Resource for Reconstructing Transcriptional Regulatory Networks from Chip-Seq Experiments in Plants. Nucleic Acids Res. 47, D1155–D1163. 10.1093/nar/gky1081 30395277 PMC6323957

[B10] Demura T. Fukuda H. (2007). Transcriptional Regulation in wood Formation. Trends Plant Sci. 12, 64–70. 10.1016/j.tplants.2006.12.006 17224301

[B11] Do C.-T. Pollet B. Thévenin J. Sibout R. Denoue D. Barrière Y. (2007). Both caffeoyl Coenzyme A 3-O-Methyltransferase 1 and Caffeic Acid O-Methyltransferase 1 Are Involved in Redundant Functions for Lignin, Flavonoids and Sinapoyl Malate Biosynthesis in Arabidopsis. Planta 226, 1117–1129. 10.1007/s00425-007-0558-3 17594112

[B12] Gallego-Giraldo L. Shadle G. Shen H. Barros-Rios J. Fresquet Corrales S. Wang H. (2016). Combining Enhanced Biomass Density with Reduced Lignin Level for Improved Forage Quality. Plant Biotechnol. J. 14, 895–904. 10.1111/pbi.12439 26190611 PMC11388942

[B13] Geraldes A. Pang J. Thiessen N. Cezard T. Moore R. Zhao Y. (2011). SNP Discovery in Black cottonwood (*Populus trichocarpa*) by Population Transcriptome Resequencing. Mol. Ecol. Resour. 11, 81–92. 10.1111/j.1755-0998.2010.02960.x 21429165

[B14] Gui J. Shen J. Li L. (2011). Functional Characterization of Evolutionarily Divergent 4-Coumarate:Coenzyme A Ligases in Rice. Plant Physiol. 157, 574–586. 10.1104/pp.111.178301 21807887 PMC3192572

[B15] Han X.-y. Mao L.-c. Lu W.-j. Tao X.-y. Wei X.-p. Luo Z.-s. (2018). Abscisic Acid Induces Differential Expression of Genes Involved in Wound-Induced Suberization in Postharvest Tomato Fruit. J. Integr. Agric. 17, 2670–2682. 10.1016/S2095-3119(18)62142-2

[B16] Herrero J. Fernández-Pérez F. Yebra T. Novo-Uzal E. Pomar F. Pedreño M. Á. (2013). Bioinformatic and Functional Characterization of the Basic Peroxidase 72 from *Arabidopsis thaliana* Involved in Lignin Biosynthesis. Planta 237, 1599–1612. 10.1007/s00425-013-1865-5 23508663

[B17] Hu D. Liu X. B. She H. Z. Gao Z. Ruan R. W. Wu D. Q. (2017). The Lignin Synthesis Related Genes and Lodging Resistance of *Fagopyrum Esculentum* . Biol. Plant 61, 138–146. 10.1007/s10535-016-0685-4

[B18] Jahan M. S. Haider M. M. Rahman M. Biswas D. Misbahuddin M. Mondal G. K. (2011). Chemical Pulping: Evaluation of Rubber wood (Hevea Brasiliensis) as a Raw Material for Kraft Pulping. Nord. Pulp Pap. Res. J. 26, 258–262. 10.3183/npprj-2011-26-03-p258-262

[B19] Kim Y.-H. Kim C. Y. Song W.-K. Park D.-S. Kwon S.-Y. Lee H.-S. (2008). Overexpression of Sweetpotato *Swpa4* Peroxidase Results in Increased Hydrogen Peroxide Production and Enhances Stress Tolerance in Tobacco. Planta 227, 867–881. 10.1007/s00425-007-0663-3 18224366

[B20] Lertpanyasampatha M. Viboonjun U. Kongsawadworakul P. Chrestin H. Narangajavana J. (2014). Differential Expression of microRNAs and Their Targets Reveals a Possible Dual Role in Physiological Bark Disorder in Rubber Tree. J. Plant Physiol. 171, 1117–1126. 10.1016/j.jplph.2014.05.001 24973583

[B21] Li C. Ma X. Yu H. Fu Y. Luo K. (2018). Ectopic Expression of *PtoMYB74* in Poplar and Arabidopsis Promotes Secondary Cell Wall Formation. Front. Plant Sci. 9, 1–13. 10.3389/fpls.2018.01262 30364214 PMC6191708

[B22] Li C. Wang X. Ran L. Tian Q. Fan D. Luo K. (2015). PtoMYB92 Is a Transcriptional Activator of the Lignin Biosynthetic Pathway during Secondary Cell Wall Formation inPopulus Tomentosa. Plant Cel Physiol 56, 2436–2446. 10.1093/pcp/pcv157 26508520

[B23] Li H. Qin Y. Xiao X. Tang C. (2011). Screening of Valid Reference Genes for Real-Time RT-PCR Data Normalization in *Hevea Brasiliensis* and Expression Validation of a Sucrose Transporter Gene HbSUT3. Plant Sci. 181, 132–139. 10.1016/j.plantsci.2011.04.014 21683878

[B24] Li H. Yang Y. Wang Z. Guo X. Liu F. Jiang J. (2016). *BpMADS12* Gene Role in Lignin Biosynthesis of *Betula Platyphylla Suk* by Transcriptome Analysis. J. For. Res. 27, 1111–1120. 10.1007/s11676-016-0229-y

[B25] Li X. Yang X. Wu H. X. (2013). Transcriptome Profiling of Radiata pine Branches Reveals New Insights into Reaction wood Formation with Implications in Plant Gravitropism. BMC Genomics 14, 768. 10.1186/1471-2164-14-768 24209714 PMC4046691

[B26] Liu B. Liu J. Yu J. Wang Z. Sun Y. Li S. (2021). Transcriptional Reprogramming of Xylem Cell wall Biosynthesis in Tension wood. Plant Physiol. 186, 250–269. 10.1093/plphys/kiab038 33793955 PMC8154086

[B27] Liu J. Shi C. Shi C.-C. Li W. Zhang Q.-J. Zhang Y. (2020). The Chromosome-Based Rubber Tree Genome Provides New Insights into Spurge Genome Evolution and Rubber Biosynthesis. Mol. Plant 13, 336–350. 10.1016/j.molp.2019.10.017 31838037

[B28] Liu Q. Luo L. Zheng L. (2018). Lignins: Biosynthesis and Biological Functions in Plants. Int. J. Mol Sci. 19, 335. 10.3390/ijms19020335 PMC5855557 29364145

[B29] Ohtani M. Demura T. (2019). The Quest for Transcriptional Hubs of Lignin Biosynthesis: beyond the NAC-MYB-Gene Regulatory Network Model. Curr. Opin. Biotechnol. 56, 82–87. 10.1016/j.copbio.2018.10.002 30390602

[B30] Pramoolkit P. Lertpanyasampatha M. Viboonjun U. Kongsawadworakul P. Chrestin H. Narangajavana J. (2014). Involvement of Ethylene-Responsive microRNAs and Their Targets in Increased Latex Yield in the Rubber Tree in Response to Ethylene Treatment. Plant Physiol. Biochem. 84, 203–212. 10.1016/j.plaphy.2014.09.016 25289520

[B31] Priyadarshan P. M. (2017). Refinements to *Hevea* Rubber Breeding. Tree Genet. Genomes 13, 1–17. 10.1007/s11295-017-1101-8

[B32] Schmittgen T. D. Livak K. J. (2008). Analyzing Real-Time PCR Data by the Comparative CT Method. Nat. Protoc. 3, 1101–1108. 10.1038/nprot.2008.73 18546601

[B33] Severo E. T. D. Calonego F. W. Sansígolo C. A. Bond B. (2016). Changes in the Chemical Composition and Decay Resistance of Thermally-Modified *Hevea Brasiliensis* Wood. PLoS One 11, e0151353. 10.1371/journal.pone.0151353 26986200 PMC4795606

[B34] Shannon P. Markiel A. Ozier O. Baliga N. S. Wang J. T. Ramage D. (2003). Cytoscape: A Software Environment for Integrated Models of Biomolecular Interaction Networks. Genome Res. 13, 2498–2504. 10.1101/gr.1239303 14597658 PMC403769

[B35] Sun Y. Jiang C. Jiang R. Wang F. Zhang Z. Zeng J. (2021). A Novel NAC Transcription Factor from *Eucalyptus*, EgNAC141, Positively Regulates Lignin Biosynthesis and Increases Lignin Deposition. Front. Plant Sci. 12, 1–10. 10.3389/fpls.2021.642090 PMC8061705 33897732

[B36] Teoh Y. P. Don M. M. Ujang S. (2011). Assessment of the Properties, Utilization, and Preservation of Rubberwood (*Hevea Brasiliensis*): A Case Study in Malaysia. J. Wood Sci. 57, 255–266. 10.1007/s10086-011-1173-2

[B51] Thimm O. BlȨsing O. Gibon Y. Nagel A. Meyer S. KrȨger P. (2020). MAPMAN: A user-driven tool to display genomics data sets onto diagrams of metabolic pathways and other biological processes. Plant J. 37, 914–939. 10.1111/j.1365-313X.2004.02016.x 14996223

[B37] Tian Q. Wang X. Li C. Lu W. Yang L. Jiang Y. (2013). Functional Characterization of the Poplar R2R3-MYB Transcription Factor PtoMYB216 Involved in the Regulation of Lignin Biosynthesis during Wood Formation. PLoS One 8, e76369. 10.1371/journal.pone.0076369 24204619 PMC3810269

[B38] Vanholme R. Morreel K. Ralph J. Boerjan W. (2008). Lignin Engineering. Curr. Opin. Plant Biol. 11, 278–285. 10.1016/j.pbi.2008.03.005 18434238

[B39] Wagner A. Tobimatsu Y. Phillips L. Flint H. Torr K. Donaldson L. (2011). *CCoAOMT* Suppression Modifies Lignin Composition in *Pinus Radiata* . Plant J. 67, 119–129. 10.1111/j.1365-313X.2011.04580.x 21426426

[B40] Wang Y.-H. Wu X.-J. Sun S. Xing G.-M. Wang G.-L. Que F. (2018). DcC4H and DcPER Are Important in Dynamic Changes of Lignin Content in Carrot Roots under Elevated Carbon Dioxide Stress. J. Agric. Food Chem. 66, 8209–8220. 10.1021/acs.jafc.8b02068 29980166

[B41] Wang Y.-X. Teng R.-M. Wang W.-L. Wang Y. Shen W. Zhuang J. (2019). Identification of Genes Revealed Differential Expression Profiles and Lignin Accumulation during Leaf and Stem Development in tea Plant (*Camellia Sinensis* (L.) O. Kuntze). Protoplasma 256, 359–370. 10.1007/s00709-018-1299-9 30121729

[B42] Wang Y. Song F. Zhu J. Zhang S. Yang Y. Chen T. (2017). GSA: Genome Sequence Archive *. Genomics, Proteomics & Bioinformatics 15, 14–18. 10.1016/j.gpb.2017.01.001 PMC5339404 28387199

[B43] Whetten R. Sederoff R. (1995). Lignin Biosynthesis. Plant Cell 7, 1001–1013. 10.1105/tpc.7.7.1001 12242395 PMC160901

[B44] Xiao Y. Yi F. Ling J. Yang G. Lu N. Jia Z. (2020). Genome-wide Analysis of lncRNA and mRNA Expression and Endogenous Hormone Regulation during Tension wood Formation in *Catalpa Bungei* . BMC Genomics 21, 1-16. 10.1186/s12864-020-07044-5 PMC7487903 32891118

[B45] Xiong S. M. Zuo X. F. Zhu Y. Y. (2005). Determination of Cellulose, Hemi-Cellulose and Lignin in Rice Hull. Cereal Feed Ind. 8, 40–41.

[B46] Yan B. Zhang Z. Zhang P. Zhu X. Jing Y. Wei J. (2019). Nitric Oxide Enhances Resistance against Black Spot Disease in Muskmelon and the Possible Mechanisms Involved. Scientia Horticulturae 256, 108650. 10.1016/j.scienta.2019.108650

[B47] You T.-T. Mao J.-Z. Yuan T.-Q. Wen J.-L. Xu F. (2013). Structural Elucidation of the Lignins from Stems and Foliage of *Arundo donax* Linn. J. Agric. Food Chem. 61, 5361–5370. 10.1021/jf401277v 23646880

[B48] Zeng J.-k. Li X. Zhang J. Ge H. Yin X.-r. Chen K.-s. (2016). Regulation of Loquat Fruit Low Temperature Response and Lignification Involves Interaction of Heat Shock Factors and Genes Associated with Lignin Biosynthesis. Plant Cel Environ. 39, 1780–1789. 10.1111/pce.12741 27006258

[B49] Zhao Q. Dixon R. A. (2011). Transcriptional Networks for Lignin Biosynthesis: More Complex Than We Thought? Trends Plant Sci. 16, 227–233. 10.1016/j.tplants.2010.12.005 21227733

[B50] Zhong R. Ye Z.-H. (2009). Transcriptional Regulation of Lignin Biosynthesis. Plant Signaling Behav. 4, 1028–1034. 10.4161/psb.4.11.9875 PMC2819510 19838072

