# Supplementary material for: RNA Sequencing Reveals Phenylpropanoid Biosynthesis Genes and Transcription Factors for Hevea brasiliensis Reaction Wood Formation
Source: Front Genet. 2021 Oct 29;12:763841. doi: 10.3389/fgene.2021.763841 (PMC8585928; doi:10.3389/fgene.2021.763841)
Supplement: Supplementary file 2 [file Table1.DOCX]

**Supplementary Table 1: R code used to calculate the Pearson correlation coefficients**

setwd(" ")

a=read.table(" ",sep="\t",head=T)

a

Gene<-c(" ")

data.frame(a,row.names=1)

t1<-t(data.frame(a,row.names=1))

t1

t2<-as.data.frame(t1,row.names=F)

t2

t3<-as.data.frame(cbind(Gene,t2))

t3

library(Formula)

library(Hmisc)

head(t2)

mat=matrix(ncol=4,nrow=sum(1:(ncol(t2)-1)))

m=1

for(i in 2:(ncol(t2)-1)){

for(j in (i+1):ncol(t2)){

mat[m,2]=names(t2)[j]

mat[m,1]=names(t2)[i]

mat[m,3]=cor(t2[,i],t2[,j],method="pearson")

w=rcorr(t2[,i],t2[,j],type="pearson")

mat[m,4]=w$P[1,2]

m=m+1

}

}

colnames(mat)<-c("gene1","gene2","correlation_coefficient","pvalue")

write.table(mat,sep="\t","organ-p-value.xls",col.names=TRUE,row.names = FALSE)
